# Central obesity and its association with retinal age gap: insights from the UK Biobank study

**DOI:** 10.1038/s41366-023-01345-x

**Published:** 2023-07-25

**Authors:** Ruiye Chen, Junyao Zhang, Xianwen Shang, Wei Wang, Mingguang He, Zhuoting Zhu

**Affiliations:** 1 Department of Ophthalmology, Guangdong Academy of Medical Sciences, Guangdong Provincial People’s Hospital, Guangzhou, China; 2 Centre for Eye Research Australia; Ophthalmology, University of Melbourne, Melbourne, VIC Australia; 3 Ophthalmology, Department of Surgery, University of Melbourne, Melbourne, VIC Australia; 4 State Key Laboratory of Ophthalmology, Zhongshan Ophthalmic Center, Sun Yat-sen University, Guangzhou, China

**Keywords:** Obesity, Epidemiology

## Abstract

**Background:**

Conflicting evidence exists on the association between ageing and obesity. Retinal age derived from fundus images has been validated as a novel biomarker of ageing. In this study, we aim to investigate the association between different anthropometric phenotypes based on body mass index (BMI) and waist circumference (WC) and the retinal age gap (retinal age minus chronological age).

**Methods:**

A total of 35,550 participants with BMI, WC and qualified retinal imaging data available were included to investigate the association between anthropometric groups and retinal ageing. Participants were stratified into 7 different body composition groups based on BMI and WC (Normal-weight/Normal WC, Overweight/Normal WC, Mild obesity/Normal WC, Normal-weight/High WC, Overweight/High WC, Mild obesity/High WC, and Severe obesity/High WC). Linear regression and logistic regression models were fitted to investigate the association between the seven anthropometric groups and retinal age gap as continuous and categorical outcomes, respectively.

**Results:**

A total of 35,550 participants (55.6% females) with a mean age 56.8 ± 8.04 years were included in the study. Individuals in the Overweight/High WC, Mild obesity/High WC and Severe obesity/High WC groups were associated with an increase in the retinal age gap, compared with those in the Normal Weight/Normal WC group (β = 0.264, 95% CI: 0.105–0.424, *P* =0.001; β = 0.226, 95% CI: 0.082–0.371, *P* = 0.002; β = 0.273, 95% CI: 0.081–0.465, *P* = 0.005; respectively) in fully adjusted models. Similar findings were noted in the association between the anthropometric groups and retinal ageing process as a categorical outcome.

**Conclusion:**

A significant positive association exists between central obesity and accelerated ageing indexed by retinal age gaps, highlighting the significance of maintaining a healthy body shape.

## Introduction

Obesity is a fast-growing public health problem worldwide, with 1·1 billion adults classified as overweight or obese [1]. The World Health Organization defined obesity as body mass index (BMI) over 30 kg/m^2^, calculated as the body weight in kilograms divided by the height in meters squared. The risks of diabetes, hypertension, and dyslipidaemia increase from a BMI of about 21.0 kg/m^2^, thereby reducing life expectancy [2]. Given this, obesity has been considered a key contributor to accelerated ageing and age-related diseases [3, 4].

Although prior studies have established the prognostic value of obesity on health status, most work has relied on obesity defined by BMI [4–6]. Recently the limitations of measuring obesity based on BMI have been brought to attention. Among individuals with the same BMI, heterogeneity exists in regional body fat distribution [1]. An increased abdominal fat distribution is strongly associated with metabolic risk factors, contributing to the development of diabetes, coronary heart disease and hypertension [7, 8]. Waist circumference (WC) as an indicator for abdominal obesity has been reported as a strong risk factor for mortality in older people independent of BMI [4, 9, 10]. In light of this, inclusion of measures of central obesity such as WC in the prognostic valueof obesity has been noted [11].

Recently, our research group verified retinal age, a predicted age derived from retinal images by a deep learning algorithm, as an easily accessible ageing biomarker [12]. The retinal age could predict chronological age with an overall MAE of 3.55 years. Previous studies have shown MAEs of 3.3–5.2 years for DNA methylation clock [13, 14], 6.2–7.8 years for the transcriptome ageing clock [15, 16], 5.5–5.9 years for blood profiles [17, 18], 4.3–7.3 years of brain age [19, 20], 2.8–6.4 years for 3D facial imaging [21, 22]. The retinal age gap, defined as the difference between the retina-predicted age and chronological age is a strong predictor for mortality and age-related diseases including cardiovascular diseases, neurodegenerative diseases and kidney failure [12, 23–26], further lending credence to its value as an ageing biomarker. Therefore, using the UK Biobank cohort, our study aimed to investigate the association between different adiposity patterns defined by a combination of BMI and WC and retinal age gaps.

## Methods

### Study design and population

The UK Biobank was a large population-based study recruiting more than 500,000 UK residents aged 40–69 years across England, Scotland, and Wales between 2006 and 2010. Informed consent was obtained from all subjects. Touchscreen questionnaires on socio-demographics, lifestyle and medical history in addition to physical measurements including anthropometrics and spirometry were completed at baseline. Clinical health outcomes were determined via data linkage to hospital records or death registers. Comprehensive ophthalmic examinations were also carried out in the UK Biobank study in 2009. A 45° non-mydriatic retinal fundus imaging of the optic disc and macular were captured for each eye. The detailed UK Biobank study protocols were available elsewhere [27].

The UK Biobank was reviewed and approved by the National Information Governance Board for Health and Social Care and the NHS North West Multicenter Research Ethics Committee (11/NW/0382) and data used in the present study was accessed through the Biobank consortium (Application No: 62489). Since this is a publicly identified dataset, the Guangdong Provincial People’s Medical Research Ethics Committee waived the ethical requirement. The study was in accordance with the Helsinki declaration with informed consent from all participants.

### Measurement and definition of anthropometric groups

Body mass index (BMI) was calculated for each individual as their weight in kilograms divided by their height in meters squared and classed into four categories based on their BMI including normal/weight, 18.5–24.9 kg/m^2^; overweight, 25–29.9 kg/m^2^; mild obesity, 30–34.9 kg/m^2^; severe obesity, ≥35 kg/m^2^. Waist circumference (WC) was measured at the smallest part of the trunk using a 200-cm tape measure (SECA). High WC was determined as WC > 102 cm for men and >88 cm for women. By combining the four BMI categories with two WC categories, individuals were categorized into seven anthropometric groups: Normal-weight/Normal WC, Overweight/Normal WC, Mild obesity/Normal WC, Normal-weight/High WC, Overweight/High WC, Mild obesity/High WC, and Severe obesity/High WC. Since only 12 people with severe obesity had normal WC, Severe obesity was not further divided by WC.

### Deep learning model for age prediction

A total of 80,169 images from 46,969 participants met the image quality criteria and were included in the analysis. Consistent with previous studies for age prediction [19], a total of 19,200 retinal fundus images from 11,052 healthy participants at baseline were fed into the deep learning (DL) model. If available, images from both eyes were used to maximize the data volume for the development and validation of DL model. In the testing dataset, the DL model was capable of accurately predicting age with a Pearson correlation coefficient of 0.80 (*P* < 0.001) and an overall mean absolute error (MAE) of 3.55 years. The protocols on the development and validation of this model have been described in detail elsewhere [12].

### Retinal age gap

Retinal age gap was defined as the difference between the retinal age derived from fundus images and the chronological age. The distribution of retinal age gap is showed in Supplementary Fig. S1. We then transformed the retinal age gap to a binary variable where the upper 50% quantile was considered to be accelerated retinal ageing, and the lower 50% quantile was considered to be non-accelerated retinal ageing.

### Covariates

Covariates in the present analyses included baseline age (continuous, years), sex (female/male), ethnicity (white and others), Townsend deprivation indices (continuous), systolic blood pressure (continuous, mmHg), education attainment (college/university/above or others), smoking status (never or former/current), alcohol drinking status (never or former/current), physical activity level (above moderate/vigorous recommendation or not), and history of diabetes, cardiovascular diseases or hyperlipidemia (yes or no). Townsend deprivation indices was a proxy measure of socioeconomic status based on the postcode.

Diabetes mellitus was defined as any record of self-reported or doctor-diagnosed diabetes mellitus, or blood HbA1c level ≥48 mmol/L or the use of anti-hyperglycaemic medications or insulin. CVD was determined via data linkage to hospital admission data and death registry records according to the International Classification of Diseases edition 10 (ICD-10) and the International Classification of Diseases edition 9 (ICD-9). Hyperlipidemia was defined as any record of self-reported high cholesterol, or the use of lipid lowering drugs or blood total cholesterol level ≥6.21 mmol/L.

### Statistical analysis

The baseline characteristics stratified by the seven anthropometric groups and the retinal ageing process were reported using means and standard deviations (SDs) for continuous variables or numbers and proportions for categorical variables. Chi square test and One-Way ANOVA were used to compare categorical or continuous variables. Age, sex, and ethnicity-adjusted logistic regression models were used to estimate the association between baseline characteristics and retinal ageing. We used linear regression models when exploring the association between obesity phenotypes and retinal age gap as a continuous outcome. We then used logistic regression model when exploring the association between obesity phenotypes and retinal age gap as a categorical outcome. Model I adjusted for age, sex and ethnicity; Model II additionally adjusted for Townsend, systolic blood pressure, education attainment, drinking status, smoking, physical activities, history of diabetes, cardiovascular diseases and hyperlipidemia. Regression coefficient (β) or odds ratio (OR) with their 95% confidence intervals (CIs) were reported for continuous or categorical outcomes, respectively. A two-sided *p* value of <0.05 indicated statistical significance. Analyses were performed using Stata (version 13, StataCorp, Texas, USA).

## Results

### Study sample

**Fig. 1:**
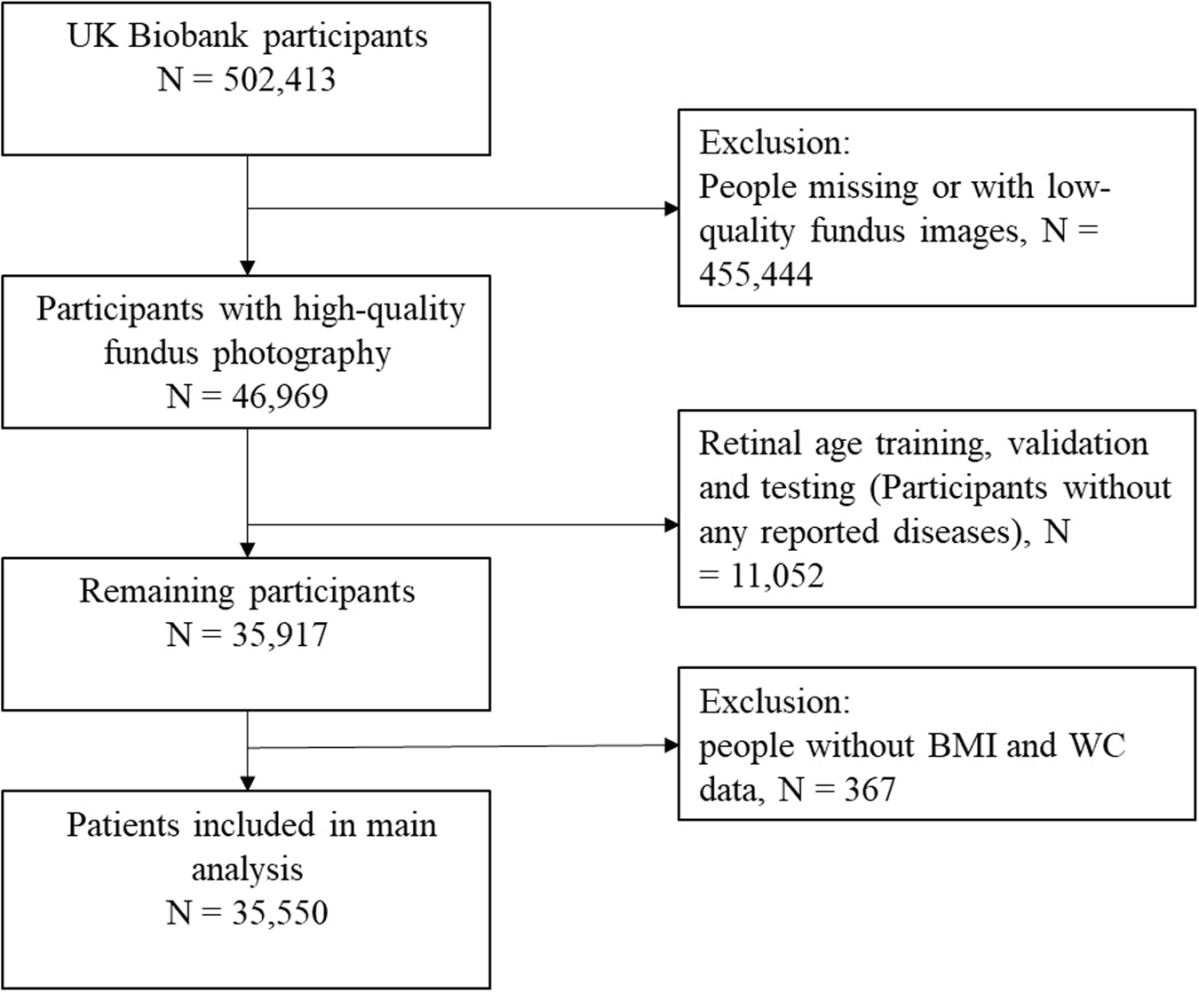
Flow chart of the included participants. People were excluded missing or with low-quality fundus images (*N* = 455,444). Participants whose fundus images were used for age prediction model training and validation (*N* = 11,052), and those without sufficient BMI and WC data (*N* =  367) were also excluded. A total of 35,550 participants were included in the final analysis.

Figure 1 shows the overall workflow of selecting participants. A total of 35,550 participants (55.6% females) with a mean age 56.8 ± 8.04 years were included in the study. Table 1 depicts the baseline characteristics of the participants stratified by seven anthropometric groups. Mean age among the seven groups was similar (55.9–58.8 years of age), with participants from the Overweight/High WC on average the oldest (58.8 ± 7.39 years). Female made up a significant proportion in the Normal weight/High WC group (217/221, 98.2%). Compared to participants in the Severe obesity/High WC group, the participants in the Normal weight/ Normal WC group are more likely to be white (93.6%), females (66.2%), non-smokers (59.2%), with a college/university education (42.5%), higher socioeconomic status, and no history of diabetes (97.6%), cardiovascular disease and hyperlipidemia (60.2%) (all *P* < 0.001).

**Table 1:** Baseline characteristics of the participants by anthropometric groups.

Baseline Characteristics	Total	Anthropometric Groups							*P* value
		Normal-weight/Normal WC	Overweight/Normal WC	Mild obesity/Normal WC	Normal-weight/High WC	Overweight/High WC	Mild obesity/High WC	Severe obesity/High WC	
*N*	35,550	11,043	11,410	1262	221	3747	5249	2618	
Age, mean (SD), yrs	56.8 (8.04)	56.0 (8.20)	56.8 (8.11)	55.9 (8.18)	58.7 (7.23)	58.8 (7.39)	57.4 (7.75)	56.1 (7.86)	**<0.001**
Gender, No. (%)									
Female	19,759 (55.6)	7305 (66.2)	4769 (41.8)	495 (39.2)	217 (98.2)	2516 (67.1)	2789 (53.1)	1668 (63.7)	**<0.001**
Male	15,791 (44.4)	3738 (33.9)	6641 (58.2)	767 (60.8)	4 (1.81)	1231 (32.9)	2460 (46.9)	950 (36.3)	
Ethnicity, NO. (%)									
White	33,154 (93.3)	10,340 (93.6)	10,654 (93.4)	1142 (90.5)	213 (96.4)	3507 (93.6)	4887 (93.1)	2411 (92.1)	**<0.001**
Others	2396 (6.74)	703 (6.37)	756 (6.63)	120 (9.51)	8 (3.62)	240 (6.41)	362 (6.90)	207 (7.91)	
Deprivation index, mean (SD)	−1.09 (2.95)	−1.22 (2.89)	−1.24 (2.88)	−1.06 (3.04)	−1.16 (2.71)	−1.11 (2.95)	−0.82 (3.09)	−0.45 (3.16)	**<0.001**
SBP, mean (SD), mmHg	137.7 (18.5)	132.7 (19.0)	138.9 (18.0)	140.4 (17.5)	135.9 (19.7)	140.0 (18.1)	141.3 (17.1)	141.6 (17.3)	**<0.001**
Education level, No. (%)									
College/university	12,336 (34.7)	4690 (42.5)	3996 (35.0)	376 (29.8)	74 (33.5)	1080 (28.8)	1460 (27.8)	660 (25.2)	**<0.001**
Others	23,214 (65.3)	6353 (57.5)	7414 (65.0)	886 (70.2)	147 (66.5)	2667 (71.2)	3789 (72.2)	1958 (74.8)	
Smoking status, No. (%)									
Never	19,606 (55.4)	6515 (59.2)	6287 (55.4)	668 (53.1)	123 (55.7)	1933 (51.8)	2660 (51.1)	1420 (54.7)	**<0.001**
Former/current	15,767 (44.6)	4499 (40.8)	5059 (44.6)	590 (46.9)	98 (44.3)	1796 (48.2)	2551 (48.9)	1174 (45.3)	
Alcohol drinking status, No. (%)									
Never	1561 (4.40)	434 (3.94)	426 (3.75)	52 (4.13)	15 (6.79)	195 (5.22)	256 (4.90)	183 (7.02)	**<0.001**
Former/current	33,884 (95.6)	10,587 (96.1)	10,949 (96.2)	1207 (95.9)	206 (93.2)	3543 (94.8)	4970 (95.1)	2422 (93.0)	
Meeting moderate/vigorous recommendation, No. (%)									
Yes	13,191 (45.3)	3751 (40.8)	3994 (41.8)	434 (42.3)	85 (49.4)	1548 (51.4)	2155 (51.9)	1224 (60.9)	**<0.001**
No	15,925 (54.7)	5433 (59.2)	5568 (58.2)	592 (57.7)	87 (50.6)	1465 (48.6)	1995 (48.1)	785 (39.1)	
History of diabetes, No. (%)									
No	33,224 (93.5)	10,779 (97.6)	10,874 (95.3)	1172 (92.9)	215 (97.3)	3451 (92.1)	4643 (88.5)	2090 (79.8)	**<0.001**
Yes	2326 (6.54)	264 (2.39)	536 (4.70)	90 (7.13)	6 (2.71)	296 (7.90)	606 (11.5)	528 (20.2)	
History of cardiovascular diseases, No. (%)									
No	33,473 (96.0)	10,627 (97.4)	10,753 (96.1)	1173 (94.9)	213 (99.1)	3515 (95.8)	4799 (93.9)	2393 (93.9)	**<0.001**
Yes	1407 (4.03)	286 (2.62)	436 (3.90)	63 (5.10)	2 (0.93)	153 (4.17)	313 (6.12)	154 (6.05)	
History of hyperlipidemia, No. (%)									
No	18,385 (51.7)	6650 (60.2)	5895 (51.7)	615 (48.7)	104 (47.1)	1608 (42.9)	2259 (43.0)	1254 (47.9)	**<0.001**
Yes	17,165 (48.3)	4393 (39.8)	5515 (48.3)	647 (51.3)	117 (52.9)	2139 (57.1)	2990 (57.0)	1364 (52.1)	

*SD* standard deviation, *SBP* systolic blood pressure.

Bold values indicates statistical significant P values (*P* < 0.05).

### Baseline characteristics associated with retinal ageing

Table 2 shows the baseline characteristics of participants stratified by accelerated and non-accelerated retinal ageing. The logistic regression models showed that after adjusting for age, sex, and ethnicity, Townsend (OR = 1.02; 95% CI: 1.01–1.03), systolic blood pressure (OR = 1.00; 95% CI: 1.00–1.00), ex/current smoking status (OR = 1.18; 95% CI: 1.12–1.24), meeting exercise recommendation(OR = 0.91; 95%CI: 0.86–0.96), history of diabetes (OR = 1.62; 95% CI:1.47–1.79) and history of hyperlipidemia (OR = 1.11; 95% CI: 1.05–1.17) were significantly associated with accelerated retinal ageing (*P* < 0.05, Table 2).

**Table 2:** Baseline characteristics of participants stratified by accelerated and non-accelerated retinal ageing.

Baseline Characteristics	Non-accelerated Retinal Ageing	Accelerated Retinal Ageing	OR (95%CI)^a^
*N*			
Age, mean (SD), yrs	61.2 (5.98)	52.3 (7.32)	**0.83(0.83–0.83)**^**b**^
Gender, No. (%)			
Female	9469 (53.3)	10,290 (57.9)	1 [reference]
Male	8306 (46.7)	7485 (42.1)	**0.86(0.82–0.90)**^**b**^
Ethnicity, No. (%)			
White	16,740 (94.2)	16,414 (92.3)	1[reference]
Others	1035 (5.82)	1361 (7.66)	**0.54(0.48–0.60)**^**b**^
Deprivation index, mean (SD)	−1.33 (2.84)	−0.85 (3.05)	**1.02(1.01–1.03)**^**b**^
SBP, mean (SD), mmHg	140.9 (18.6)	134.5 (17.7)	**1.00(1.00–1.00)**^**b**^
Education level, No. (%)			
College/university	5639 (31.7)	6697 (37.7)	1[reference]
Others	12,136 (68.3)	11,078 (62.3)	0.99(0.94–1.05)
Smoking status, No. (%)			
Never	9620 (54.4)	9986 (56.5)	1[reference]
Former/current	8062 (45.6)	7705 (43.5)	**1.18(1.12–1.24)**^**b**^
Alcohol drinking status, No. (%)			
Never	786 (4.43)	775 (4.38)	1[reference]
Former/current	16,953 (95.6)	16,931 (95.6)	0.89(0.79-1.01)
Meeting moderate/vigorous recommendation, No. (%)			
Yes	6256 (43.4)	6,935 (47.1)	**0.91(0.86–0.96)**^**b**^
No	8147 (56.6)	7778 (52.9)	1[reference]
History of Diabetes, No. (%)			
No	16,613 (93.5)	16,611 (93.5)	1[reference]
Yes	1162 (6.54)	1164 (6.55)	**1.62(1.47–1.79)**^**b**^
History of cardiovascular diseases, No. (%)			
No	16,478 (95.0)	16,995 (97.0)	1[reference]
Yes	875 (5.04)	532 (3.04)	1.12(0.98–1.27)
History of hyperlipidemia, No. (%)			
No	8010 (45.1)	10,375 (58.4)	1[reference]
Yes	9765 (54.9)	7400 (41.6)	**1.11(1.05–1.17)**^**b**^

*SD* standard deviation, *SBP* systolic blood pressure.

^a^Adjusted for age, sex, and ethnicity.

^b^*P* < 0.05.

Bold values indicates statistical significant P values (*P*  <  0.05).

### Anthropometric groups and retinal age gap

As shown in Table 3, after adjusting for age, gender and ethnicity, individuals in the Overweight/High WC, Mild obesity/High WC and Severe obesity/High WC groups were associated with an increase in the retinal age gap, compared with those in the Normal Weight/Normal WC group (β = 0.333, 95% CI: 0.191–0.474, *P* < 0.001; β = 0.383, 95% CI: 0.257–0.509; *P* < 0.001; β = 0.440, 95% CI: 0.278–0.602, *P* < 0.001, respectively). The associations between Overweight/High WC, Mild obesity/High WC and Severe obesity/High WC groups and retinal age gap remained significant after adjustments for additional confounding factors (β = 0.264, 95% CI: 0.105-0.424, *P* = 0.001; β = 0.226, 95% CI: 0.082–0.371, *P* = 0.002; β = 0.273, 95% CI: 0.081–0.465, *P* = 0.005; respectively). Participants in the Overweight/Normal WC, Mild obesity/Normal WC or Normal-weight/High WC groups showed no significant associations with retinal age gaps.

**Table 3:** Association between anthropometric groups with retinal age gaps as a continuous outcome.

	Model I		Model II	
Anthropometric Groups	β (95% CI)	*P* value	β (95% CI)	*P* value
Normal-weight/Normal WC	Reference	-	Reference	-
Overweight/Normal WC	0.038 (-0.063–0.140)	0.462	0.041 (-0.071–0.154)	0.470
Mild obesity/Normal WC	0.058 (-0.164–0.280)	0.612	0.036 (-0.213–0.285)	0.776
Normal-weight/High WC	0.220 (-0.288–0.727)	0.396	-0.167 (-0.744–0.409)	0.569
Overweight/High WC	0.333 (0.191–0.474)	**<0.001**	0.264 (0.105–0.424)	**0.001**
Mild obesity/High WC	0.383 (0.257–0.509)	**<0.001**	0.226 (0.082–0.371)	**0.002**
Severe obesity/High WC	0.440 (0.278–0.602)	**<0.001**	0.273 (0.081–0.465)	**0.005**

*WC* waist circumference, *β* regression coefficient.

Model I adjusted for age, gender, and ethnicity;

Mode II adjusted for age, gender, ethnicity, townsend, education attainment, drinking status, smoking, physical activities, systolic blood pressure, history of diabetes and cardiovascular diseases and hyperlipidemia.

Bold values indicates statistical significant P values (*P*  <  0.05).

Similar findings were noted in the association between the anthropometric groups and retinal ageing process as a categorical outcome, as shown in Table 4. The age, sex and ethnicity-adjusted model showed that participants in the Overweight/High WC, Mild obesity/High WC and Severe obesity/High WC groups were associated with a higher risk of accelerated ageing compared the Normal Weight/Normal WC group (OR = 1.18, 95% CI: 1.08–1.29, *P* < 0.001; OR = 1.20, 95% CI: 1.11–1.30, *P* < 0.001; OR = 1.27, 95% CI: 1.15–1.41, *P* < 0.001; respectively). The associations remained significant in the fully adjusted model (OR = 1.14, 95% CI: 1.03-1.26, *P* = 0.012; OR = 1.10, 95% CI: 1.00–1.21, *P* = 0.044; OR = 1.20, 95% CI: 1.06–1.36, *P* = 0.003; respectively).

**Table 4:** Association between anthropometric groups with retinal ageing as a category outcome.

	Model I		Model II	
Anthropometric Groups	OR (95% CI)	*P* Value	OR (95% CI)	*P* Value
Normal-weight/ Normal WC	Reference [1]	-	Reference [1]	-
Overweight/Normal WC	1.02 (0.96–1.09)	0.545	1.01 (0.94–1.09)	0.795
Mild obesity/Normal WC	0.98 (0.85–1.13)	0.760	0.98 (0.84–1.15)	0.824
Normal-weight/High WC	1.36 (0.99–1.86)	0.055	1.16 (0.81–1.66)	0.425
Overweight/High WC	1.18 (1.08–1.29)	**<0.001**	1.14 (1.03–1.26)	**0.012**
Mild obesity/High WC	1.20 (1.11–1.30)	**<0.001**	1.10 (1.00–1.21)	**0.044**
Severe obesity/High WC	1.27 (1.15–1.41)	**<0.001**	1.20 (1.06–1.36)	**0.003**

*WC* waist circumference, *OR* odd ratio.

Model I adjusted for age, gender, and ethnicity.

Mode II adjusted for age, gender, ethnicity, Townsend, education attainment, drinking status, smoking, physical activities, systolic blood pressure, history of diabetes and cardiovascular diseases and hyperlipidemia.

Bold values indicates statistical significant P values (*P*  < 0.05).

## Discussion

This population-based cohort study found a significant positive association between central obesity and retinal age gaps from seven different adiposity patterns. Severe obesity/High WC group showed the highest increased risk of accelerated retinal ageing compared with those in the Normal weight/Normal WC group. Taken together, these findings highlight abdominal adiposity as a clinical sign of accelerating ageing.

Our study is the first to reveal the association between central obesity and biological age indexed by retinal age gap. Previous studies have investigated the associations between obesity indices and ageing biomarkers. Telomere length and DNA methylation are two well established ageing biomarkers [28, 29]. Several studies found both BMI and WC were inversely associated with leukocyte telomere length in adults [30–34]. Accelerated aging assessed by DNA methylation were also associated with increased BMI and WC [35, 36]. Recently, brain age derived from brain imaging has been verified as a non-invasive ageing biomarker [19]. Obesity by BMI was associated with accelerated brain ageing while weight loss may slow the pace of brain ageing [37, 38].

Furthermore, our finding has provided a possible explanation for obesity paradox. The obesity paradox is a term that has been used to describe a seemingly paradoxical phenomenon in which individuals who are overweight or obese appear to have a lower risk of mortality compared to those who are of normal weight or underweight [39]. Most of these studies used BMI as obesity indicators [40, 41]. BMI represents the sum of fat-mass index (FMI) and fat-free mass index (FFMI) [42]. FFMI accounts for skeletal muscle mass, bone, and organs, while FMI is composed of peripheral and visceral adipose tissues [42]. These components of BMI have different roles in contributing to health status, highlight the limitations of BMI in accurately reflecting the distribution of body fat or lean mass [43]. This may affect the interpretation of weight-related health risks, as changes of BMI do not relate to a proportional and linear modification of body compartments. Thus, a slightly high BMI does not indicate poor prognosis. Our study further categorized patients with high BMI normally observed with the obesity paradox into two groups: those with high WC and those with low WC. Only patients with high body fat indicated by jointly high BMI and WC are associated with poor prognosis. Thus, the obesity paradox may be explained by the lack of discriminatory power of BMI to differentiate between fat mass and mean muscle mass.

There are several proposed mechanisms for our findings. Our results showed that subjects with high BMI as well as high WC are related to abnormal accelerated ageing. High BMI and WC indicates high visceral adipose tissues, which has been reported to secrete numerous immunomodulatory factors including resistin [44], tumor necrosis factor (TNF)-alpha and C-reactive protein [45, 46], contributing to insulin resistance and hypertriglyceridemia, ultimately accelerating the vascular ageing rate [47, 48]. Elevated oxidized LDLs were also noted to modify DNA methylation pattern or even cause cellular DNA damage, subsequently leading to cellular senescence [3, 49, 50]. However, we didn’t find accelerated ageing among individuals with overweight or mild obesity but normal WC. This could be partly explained from the protective effects of skeletal muscle within those participants [42, 43]. Muscle-derived IL-6 has been found to inhibit the endotoxin-induced increase in circulating levels of TNF-alpha in healthy individuals [51]. Muscle could also produce and releases anti-inflammatory cytokines such as IL-10 and TGF-β, improving the peripheral insulin sensitivity via the activation of AMPK [52].

Our findings have several implications. Firstly, our findings further consolidated retinal age as a robust marker of ageing and overall health status. Secondly, measures of central obesity such as waist circumference should complement BMI in clinical practice to accurately identify individuals at increased risk of adverse health outcomes. The concept of “central obesity drives accelerated ageing” emphasizes to individuals the multi-system effects of obesity and the importance of maintaining good body shape rather than solely reducing the body weight. Furthermore, the non-invasive and accessible nature of the retinal age gap makes it an acceptable marker for monitoring of ageing pace over time, providing individuals with real-time feedback.

Based on the UK Biobank study, our study had numerous strengths including its large sample size, standardized protocols and comprehensive adjustment of confounding factors. However, there are also several limitations. Firstly, the UK Biobank study is comprised of volunteers who are healthier and may not be representative of the general population. Secondly, we cannot infer causal relationships between obesity and retinal age gaps from this cross-sectional study. Finally, we are unable to fully exclude the possibility of residual confounding variables.

## Conclusion

In conclusion, our study found a significant positive association between central obesity and retinal age gaps, highlighting the value of waist circumference in the assessment and management of health risks in patients with obesity. This indicates maintaining good body shape may be crucial to reduce risk of accelerated ageing. Further studies are needed to confirm our finding.

### Supplementary information

Supplementary files

checklist

## Data Availability

The UK biobank is a publicly available database where data could be accessed through reasonable requests.

## Code availability

The code associated with this work is available upon a reasonable request to the authors.
