# Supplementary figures and images for: Central obesity and its association with retinal age gap: insights from the UK Biobank study
Source: Int J Obes (Lond). 2023 Jul 25;47(10):979–85. doi: 10.1038/s41366-023-01345-x (PMC10511312; doi:10.1038/s41366-023-01345-x)

Supplementary Figure 1. Distribution of retinal age gap in included participants.


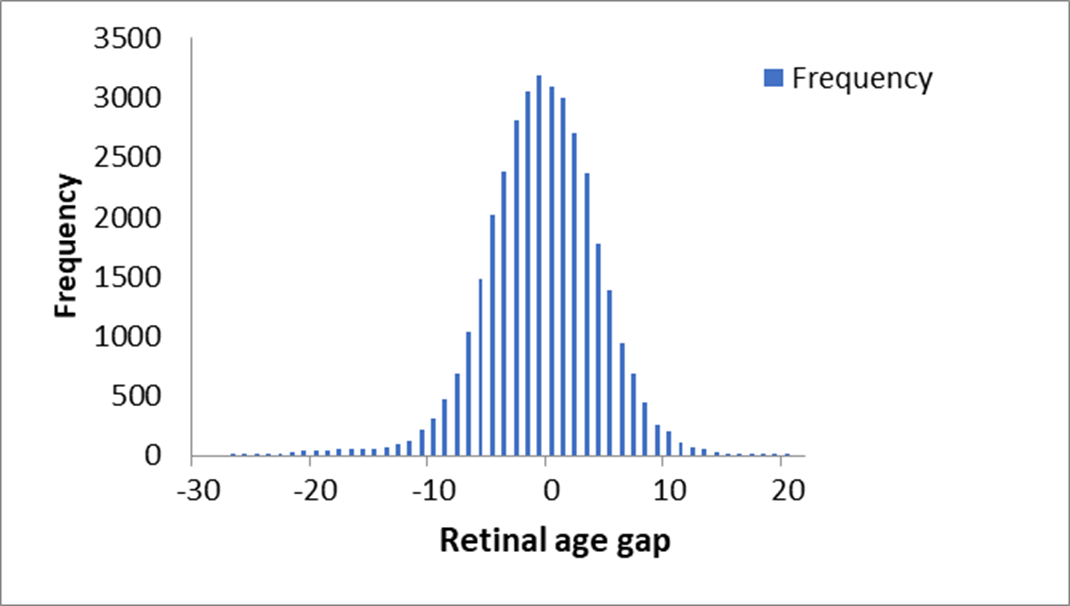

Supplement: Supplementary file 1 — Supplementary files [file 41366_2023_1345_MOESM1_ESM.docx]
